# Association of Gut Microbiota With Intestinal Ischemia/Reperfusion Injury

**DOI:** 10.3389/fcimb.2022.962782

**Published:** 2022-07-12

**Authors:** Jingyi Chen, Yu Wang, Yongxia Shi, Yongpan Liu, Chengyi Wu, Yanrong Luo

**Affiliations:** ^1^ Department of Anesthesiology, Taihe Hospital, Hubei University of Medicine, Shiyan, China; ^2^ Department of Surgical Nursing, Taihe Hospital, Hubei University of Medicine, Shiyan, China; ^3^ Physical Examination Center, Shiyan Hospital of Integrated Traditional and Western Medicine, Shiyan, China

**Keywords:** intestinal microbiota, metabolites, intestinal ischemia reperfusion, intestinal dysbiosis, biomarkers

## Abstract

Intestinal ischemia/reperfusion (II/R) is a common acute and critical condition in clinical practice with a high mortality rate. However, there is still a lack of effective prevention and treatment measures for II/R injury. The role of the gut microbiota in II/R has attracted widespread attention. Recent evidence has demonstrated that the gut microbiota plays a pivotal role in the occurrence, development, and prognosis of II/R. Therefore, maintaining the homeostasis of gut microbiota and its metabolites may be a potential strategy for the treatment of II/R. This review focuses on the importance of crosstalk between the gastrointestinal ecosystem and II/R to highlight II/R-induced gut microbiota signatures and potential applications of microbial-based therapies in II/R. This will also provide potentially effective biomarkers for the prediction, diagnosis and treatment of II/R.

## Introduction

Intestinal ischemia/reperfusion (II/R) injury is a clinical emergency with high morbidity and mortality due to a dramatic reduction in intestinal blood supply, which is associated with mesenteric artery thrombosis, strangulated ileus, trauma, abdominal aortic aneurysm surgery, intestinal transplantation and other clinical conditions (Ma et al., 2020, Wu et al., 2021a). Intestinal ischemia results in cellular damage and impaired intestinal mucosal barrier function during reperfusion, leading to systemic inflammatory response syndrome, multiple organ dysfunction syndrome, and even death (Gutiérrez-Sánchez G et al., 2021). II/R will not only cause damage to the intestinal tissue itself, but also damage to distant organs. Gut microbiota is a general term for a group of microbial communities living in the body’s gut (de Wouters d’Oplinter et al., 2021; Qi et al., 2021; Hosseinkhani et al., 2021). In recent years, more and more research evidence has shown that the gut microbiota is directly related to the occurrence of various human diseases (Bhattarai et al., 2021; Cao et al., 2021; Deng et al., 2021c; Li et al., 2021; Prochazkova P et al., 2021; Rao et al., 2021). The research on the changes and potential roles of gut microbiota in II/R has rapidly increased, which has attracted widespread attention. In this review, we aimed to summarize changes and potential diagnostic, therapeutic or exacerbating roles of gut microbiota in II/R, providing a solid theoretical basis and potentially effective biomarkers for the prevention and treatment of II/R injury.

## II/R Overview and Current Dilemma

The intestinal tract plays an extremely important role in the normal life activities of the human body (Bruellman and Llorente, 2021). The gut is the core organ of human immunity, in which 60-70% of the immune cells of the human body are located in the gut to resist the invasion of foreign germs and toxins (Hou et al., 2020; Fan et al., 2021; Liu et al., 2021). In the daily diet and metabolism, the intestinal tract gathers the most important garbage and toxins in the human body, and will eventually be excreted through the feces, which is also an important channel for the excretion of more than 80% of the toxins in the human body (Zhao et al., 2021; Butt and Jenab, 2021; Chen et al., 2021). In addition, more than 97.5% of the nutrients necessary for the human body are obtained from the intestinal absorption (Tiffany et al., 2021); 95% of the core important hormones of the human body are produced by the intestinal tract, and it is currently known that the human gastrointestinal tract can secrete more than 35 kinds of hormones (Mayneris-Perxachs et al., 2020; Lin et al., 2021; Qi et al., 2021). The intestine is the second brain of the human body, with hundreds of millions of nerve cells and a complex neural network distributed. The neural network of the intestine is directly related to the brain, forming a brain-gut-bacteria axis, which affects the nervous system through the metabolism of intestinal flora (Rutsch et al., 2020; Aktar et al., 2020; Zhang et al., 2021). Furthermore, about 78% of the flora of the human body lives in the intestinal tract. The bacteria combine, restrict and depend on each other to form a micro-ecological balance, which maintains the absorption of nutrients, the discharge of toxins, the secretion of neurotransmitters and hormones, the stability of the neural network, and ensures the immunity of the human body. Therefore, health comes from the micro-ecological balance of the flora.

II/R is a common pathophysiological process of a variety of critical diseases and organ damage. It not only causes local inflammation and mucosal damage, but also systemic inflammatory response syndrome and multiple organ dysfunction syndrome. Therefore, it is of great clinical significance to protect the intestines in the perioperative period and to explore the intervention methods with clinical translation value by studying the mechanism of II/R hindgut injury and II/R extraintestinal organ (such as brain, lung) injury. The intestinal mucosal barrier is composed of mechanical barrier, chemical barrier, immune barrier and biological barrier. It is generally believed that the failure of the intestinal barrier function may lead to the longitudinal displacement of pathogenic bacteria and their metabolites from the intestinal lumen to the blood circulatory system, which in turn induces a chronic inflammatory response in the whole body, and even the failure of various organ functions. Diamine oxidase (DAO) is a highly active intracellular enzyme in the cytoplasm of human and all mammalian intestinal mucosal cells. When intestinal mucosal cells are damaged and necrotic, DAO is released into the blood, resulting in increased blood activity. Blood DAO level is a good indicator for monitoring intestinal mucosal damage (Bragg et al., 1991). D-lactic acid is a metabolite of intestinal bacteria. When the body is severely injured or infected, the intestinal mucosal ischemia and hypoxia cause the epithelial top of the intestinal mucosa to fall off, the permeability of the intestinal mucosa increases, and a large amount of D-lactic acid enters the bloodstream, while mammals do not has an enzyme system that breaks it down, so plasma D-lactic acid levels can be significantly increased (Günel et al., 1998). Therefore, the determination of D-lactic acid content in blood can reflect changes in the degree of intestinal mucosal ischemia and intestinal permeability. There are a large number of G^-^bacteria in the intestine, which can produce a large amount of endotoxin. When serious diseases such as severe trauma, burns and peritonitis occur, the permeability of the intestinal mucosa increases, the intestinal barrier function is destroyed, and endotoxin enters the blood, resulting in endotoxemia. Therefore, plasma endotoxin levels can also reflect the intestinal barrier function. At present, the detection of plasma DAO, D-lactic acid, and bacterial endotoxin levels is regarded as the main detection index of intestinal barrier function. However, there is still a lack of effective II/R diagnostic criteria and preventive measures in clinical practice.

## Gut Microbiome

Gut microbiota is the microflora that lives in the human intestine. The number of bacteria in the human intestine is 40 trillion, and the total number of genes is about 150 times the number of human genes (Parker et al., 2020; Kazemian et al., 2020; Caparrós et al., 2021). It can be seen from the data that the gut microbiota is a very large group, so the intestinal flora is also called the “second genome”, “second brain” and “gut brain” of the human body (Liu et al., 2021, Tourlousse et al., 2021). The gut flora digests food components (Coker et al., 2021; Naimi et al., 2021), synthesizes essential vitamins (Li et al., 2020; Zafar and Saier, 2021), stimulates and modulates the immune system (Han et al., 2021a, Nogal et al., 2021), eliminates pathogens (Maslanka et al., 2020; Silwal et al., 2021), clears toxins and carcinogens (de Nies et al., 2021), supports gut function. Under normal circumstances, the gut microbiota can establish a dynamic ecological balance with the host and the external environment. Once the gut microbiota is disturbed and unbalanced, it will lead to the loss of various functions of the host, such as loss of barrier function, loss of inflammation and immune function, etc., thereby causing disease (Wang et al., 2020, Han et al., 2021b). It is reported that 95% of diseases are related to gut microbiota. In addition to gastrointestinal diseases, metabolic diseases, gut microbiota is also related to various system diseases, such as nervous system (Vicentini et al., 2021; Saldana-Morales et al., 2021), respiratory system (Zheng et al., 2020; Casaro et al., 2021), cardiovascular system (Khan et al., 2021; Deng et al., 2022), tumor (Yang et al., 2020; Chung et al., 2021; Murphy et al., 2021), etc. disease related. The gut microbiota mainly interacts closely with the host through small-molecule metabolites (such as short-chain fatty acids, bile acids, tryptophan, amino acids, etc.) (Dong et al., 2020; Kumari et al., 2020; Winston and Theriot, 2020; Guzior and Quinn, 2021; Sun et al., 2021; Wu et al., 2021b), affecting its occurrence, and therefore, there is a growing need to understand the importance of the microbiota in II/R development, diagnosis, and therapy.

## II/R-Related Gut Microbial Signatures

Ischemia results in decreased blood flow and oxygen supply to the intestinal tissue, while reperfusion will promote the accumulation and burst of harmful factors such as reactive oxygen species and inflammatory factors. Changes in the gut microenvironment will inevitably lead to changes in the quantity or population of gut microbiota, and summarizing these changes will help us explore potential biomarkers for predicting and diagnosing II/R. As shown in **Table 1**, II/R leads to small intestinal bacterial overgrowth and the formation of ceramides in the jejunal vasculature, which may contribute to the intestinal permeability associated with this injury. The new discovery of ceramides in bacterial membranes represents a new opportunity to study the dynamic pathogenicity of the gut microbiome (Hoehn et al., 2016). II/R-induced changes in gut microbiota also differed in different intestinal segments. Denaturing gradient gel electrophoresis of the ileal microbiota revealed that the gut microbiota pattern changed early after II/R, with significant differences at 12 h of reperfusion, and then began to return to a normal pattern. Specific dysbiosis is characterized by proliferation of *Escherichia coli* and reduction of *Lachnospiraceae* and *Lactobacilli* (Wang et al., 2013b). Deng et al. found that the relative abundance of *Firmicutes* and *Bacteroidetes* in the ileum of mice in the II/R group was significantly increased, while the relative abundance of *Verrucobacterium* was significantly decreased (Deng et al., 2021). Colonic microbiota changed earlier, with significant differences 6 hours after reperfusion, and then began to recover. These changes were characterized by increases in *Escherichia coli* and *Prevotella oralis*, as well as proliferation of *Lactobacilli* and healing of epithelial cells (Wang et al., 2012).

**Table 1:** Intestinal ischemia/reperfusion-induced changes in gut microbiota.

Species	Location	II/R-induced changes in gut microbiota	References
Mice	Jejunum	Bacterial counts ↑	(Hoehn et al., 2016)
Rats	Ileal	*Escherichia coli* ↑ *Lachnospiraceae* and *Lactobacilli* ↓	(Wang et al., 2013a)
Rats	Colon	*Escherichia coli* and *Prevotella oralis* ↑ *Lactobacilli* ↓	(Wang et al., 2012)
Mice	Cecum	Diversity ↑ *Bacteroides vulgatus* ↑ *Parabacteroides distasonis* ↑ *Verrucomicrobia* ↓	(Deng et al., 2021b)

## The Effect of Gut Microbiota on II/R

Not only does II/R lead to changes in the gut microbiota, dysbiosis can also affect II/R. As shown in **Table 2**, depletion of gut commensals reduces B cell, Igs, and TLR expression in the gut, inhibits complement activation, and attenuates intestinal inflammation and injury after II/R (Yoshiya et al., 2011). The research has found that gut microbiota suppresses neutrophil hyperreactivity in II/R injury, while ensuring immunovigilance by enhancing neutrophil recruitment (Ascher et al., 2020). The use of antibiotics to clear the flora is an effective way to treat infections in clinical practice. Another method to correct intestinal flora disturbance is fecal microbiota transplantation. Fecal microbiota transplantation is to transplant the functional flora in the feces of healthy people into the patient’s gastrointestinal tract to rebuild new intestinal flora and achieve the treatment of intestinal and extra-intestinal diseases (Jing et al., 2021; Manrique et al., 2021; Trikha et al., 2021). Before II/R occurs, alterations in the gut microbiota may increase susceptibility to II/R through multiple mechanisms, including: (1) the expansion of pathogenic gut bacteria; (2) the activation of the immune system that produces strong pro-inflammatory response; (3) reducing the production of beneficial microbial products such as short-chain fatty acids. Hu et al. found that differences in the pre-II/R gut microbiota, especially in the abundance of *Lactobacillus murinus*, strongly influence II/R susceptibility and outcome. Moreover, the abundance of *Lactobacillus murinus* in preoperative feces of cardiopulmonary bypass patients was significantly negatively correlated with postoperative intestinal injury (Hu et al., 2022). Pretreatment of animals with *Bifidobacteria* prevented II/R-induced bacterial translocation, reduced proinflammatory cytokine release, endotoxin levels, intestinal epithelial apoptosis, disruption of tight junctions, and increased SCFA concentrations, leading to recovery microbiota and mucosal integrity (Wang et al., 2013b). Wang et al. revealed that pretreatment of animals with *Lactobacillus plantarum* completely prevented II/R-induced bacterial translocation, reduced proinflammatory cytokine release and intestinal epithelial apoptosis, thereby restoring microflora and mucosal integrity (Wang et al., 2011). Animals pretreated with *Bifidobacterium bifidum PRL2010* showed lower neutrophil recruitment in the lungs, significantly reduced bacterial translocation, and reduced transcript levels of TNFα and IL-10 in liver and kidney, while suppressing pathogenic bacteria adhesion and enhancement of the host innate immune response are one of the possible protective mechanisms exerted by probiotics (Duranti et al., 2018). Hu et al. showed that Lactobacillus murinus attenuates II/R injury in mice *via* macrophages. In addition to intestinal bacterial strains, it has recently been found that intestinal flora metabolites are also non-negligible substances in II/R (Hu et al., 2022). Deng et al al. found that gut microbial metabolite pravastatin or capsiate attenuate II/R injury and improved the survival of mice, and pravastatin or capsiate levels in the preoperative stool of patients undergoing cardiopulmonary bypass were negatively correlated with the indices of postoperative intestinal I/R injury (Deng et al., 2021a, Deng et al., 2021b).

**Table 2:** The effect of gut microbiota on intestinal ischemia/reperfusion.

Treatment	Species	Main findings	References
Antibiotic cocktail	Mice	B cells, Igs, and TLR expression in the intestine ↓ Complement activation ↓ intestinal inflammation ↓	(Yoshiya et al., 2011)
Germ-free mice	Mice	Gut Microbiota Restricts neutrophil extracellular traps	(Ascher et al., 2020)
Altered Schaedler flora	Mice	Leukocyte adherence ↓ II/R ↓	(Bayer et al., 2021)
*Bifidobacteria*	Mice	Bacterial translocation ↓ Pro-inflammatory cytokine release ↓ The levels of endotoxin ↓ Intestinal epithelial cell apoptosis ↓ Tight junction and the concentration of SCFA ↑	(Wang et al., 2013b)
*Lactobacillus plantarum*	Rats	Bacterial translocation ↓ Intestinal barrier dysfunction ↓	(Wang et al., 2011)
*Bifidobacterium bifidum PRL2010*	Mice	Neutrophil recruitment in the lungs ↓ Bacterial translocation ↓ The transcription levels of TNFalpha and IL-10 both in liver and kidneys ↓ The adhesion of pathogenic bacteria ↓	(Duranti et al., 2018)
*Lactobacillus murinus*	Mice	Intestinal injury ↓ Mouse survival ↑	(Hu et al., 2022)
Pravastatin	Mice	Intestinal injury ↓ Mouse survival ↑	(Deng et al., 2021b)
Capsiate	Mice	Intestinal epithelial cell ferroptosis ↓ Intestinal injury ↓ Mouse survival ↑	(Deng et al., 2021b)

Once II/R occurs, disturbances in the gut microbiota are exacerbated and increase the likelihood of organ failure. Microbial treatments may be potentially effective approaches to reduce the risk of II/R and improve II/R outcomes in certain patient populations, but further studies are needed to demonstrate the safety of these treatments.

## Conclusion

This review summarizes the characteristics of II/R-induced changes in gut microbiota, and also summarizes the application of gut microbiota and their metabolites in the prevention and treatment of II/R injury in recent years. Gut microbiota will also play an increasingly important role in the prediction, diagnosis and treatment of clinical II/R in the future. However, the current study of gut microbiota in II/R also has certain limitations. At present, most studies on gut microbiota focus on preclinical animal studies. How to make the results of basic research better applied to the clinic is the most pressing issue.

## Author Contributions

YW and JC contributed equally to the writing of this manuscript; YS, YRL, CW, and YPL designed the illustrations. All authors contributed to the article and approved the submitted version.

## Funding

This work was supported by grants from Shiyan Taihe Hospital Scientific Research Supporting Fund (2019SCI013 to JC).

## Conflict of Interest

The authors declare that the research was conducted in the absence of any commercial or financial relationships that could be construed as a potential conflict of interest.

## Publisher’s Note

All claims expressed in this article are solely those of the authors and do not necessarily represent those of their affiliated organizations, or those of the publisher, the editors and the reviewers. Any product that may be evaluated in this article, or claim that may be made by its manufacturer, is not guaranteed or endorsed by the publisher.
